# Education of trainees, training and fellowships for head and neck oncologic and surgical training in the UK: United Kingdom National Multidisciplinary Guidelines

**DOI:** 10.1017/S0022215116000670

**Published:** 2016-05

**Authors:** R Simo, A Robson, B Woodwards, P Niblock, P Matteucci

**Affiliations:** 1 Department of Otolaryngology – Head and Neck Surgery, Guy's and St Thomas’ Hospital NHS Foundation Trust, Guy's, King's and St Thomas’ Medical and Dental School, London, UK; 2 North Cumbria University Hospitals NHS Trust, Cumberland Infirmary, Carlisle, UK; 3 Oral and Maxillofacial Unit, North Manchester General Hospital, Manchester, UK; 4 Ninewells Hospital, Dundee, UK; 5 Hull and East Yorkshire Hospitals NHS Trust, Hull, UK

## Abstract

Since the previous edition of these guidelines, significant changes have taken place in the training and assessment of surgeons and oncologists who treat patients with head and neck cancer. For those intending to become head and neck surgeons, a fellowship in head and neck surgery is virtually mandatory. This paper summarises the current career structure to specialise in head and neck oncology and surgery in the UK.

**Recommendation:**

• Trainees applying for head and neck surgical oncology consultant posts should have completed additional training in the subspecialty.

## Introduction

Education in the practice of head and neck oncology (HNO) has been identified as one of the key challenges in the management of head and neck cancer in the 21st century. The re-structuring and shortening of the training programmes over the last two decades has promoted the creation of interface and post-specialty training dedicated fellowship posts, with the ultimate aim of improving patient care. These changes however, require a much more substantial input from trainers which ultimately impacts on patient care, but there is no doubt that better structured and dedicated time in subspecialty training is required.

## Educational principles

Training in head and neck surgical oncology (HNSO) in the UK, both in the parent specialty and for interface trainees are governed by a curriculum approved by the General Medical Council. From 2007, all surgical trainees have been required to follow the curriculum for training as set out in the Intercollegiate Surgical Curriculum project.^1^ Clinical oncology and medical oncology training programmes are supervised by the Royal College of Radiologists (RCR)^2^ and the Royal College of Physicians (RCP)^3^, respectively.

A curriculum consists of an aim, a syllabus, an assessment matrix and a process for evaluation. As far as possible, trainees should be responsible for their own learning and achieve the objectives set out in the curriculum; trainers and overseeing bodies such as deaneries and the specialist advisory committees (SAC) should facilitate the process by ensuring that standards are met and that opportunities are available.

At the beginning of a rotation, trainees should self-assess their learning needs by comparing their current level of knowledge or technical competence against what is expected of them for their stage of training as per the curriculum. Objectives are available within the syllabus on the Intercollegiate Surgical Curriculum Programme (ISCP). This will identify the gap between what they know or can do compared with what they need to know or achieve for technical competence (a learning need). From this, a learning plan or agreement can be agreed. This plan needs to be constructed using SMART objectives (Specific, Measurable, Achievable, Relevant and Timely) so that, at the end of an attachment or programme, the trainee can be assessed to ensure that objectives have been achieved. The learning plan should be recorded in the trainee's portfolio.

### Assessment

Assessment is formative or summative. Summative assessment usually takes the form of an examination (FRCS, FRCR), is high stakes (pass or fail) and usually occurs at the end of a programme or at crucial waypoints along a programme (e.g. Member of the Royal College of Surgeons (MRCS)). Formative assessment should be viewed as an assessment for learning, to identify strengths and weaknesses in a trainee's work and to highlight areas for development. Formative assessment tools usually take the form of workplace based assessments (WPBAs). These are designed to assess the essential domains of learning of knowledge, skills, professionalism and attitudes and should be viewed as developmental rather than punitive assessments.

An integral part of adult learning is the timely and regular provision of constructive feedback. This has to be used correctly to ensure it is viewed in a positive manner. Feedback should be timely, relevant and constructive, usually given to best effect in private immediately after a learning event. Provision of written and verbal feedback is an integral part of WPBAs and aids in the agreement of areas for development.

Each trainee will be awarded an Annual Record of Competency Progression certificate (ARCP). This is to ensure that there is documentary evidence to confirm that the trainee has met his or her targets for the year and is progressing satisfactorily. Annual Record of Competency Progression certificate panels are required to examine evidence of competence and increasingly this is being carried out with more structure and objectivity than was the case with the Record of In Training Assessment system. It is thus imperative that evidence to support acquisition of competency is recorded. An ARCP panel may recommend specific targets that need to be attained (ARCP 2) or an extension to training time if a trainee requires more time to progress safely (ARCP 3).

Trainees with specific needs (Trainees with Differing Needs) require skill, sensitivity and dedicated time to ensure specific personal targets for training can be agreed and met. Trainers and trainees should seek and receive support from their deanery, employing trust and SAC to ensure satisfactory progression.

## Career structure in otorhinolaryngology, head and neck surgery

Training in otorhinolaryngology, head and neck surgery (ORL-NHS) starts as part of core surgical training (CST) for two years. Entry to ST3 is by competitive interview against personal specification including successful acquisition of the Member of the Royal Colleges of Surgeons (MRCS) (ENT).

Higher surgical training lasts six years and during this time all trainees are expected to develop competence in all aspects of the specialty. Trainees should take their Intercollegiate Exam from ST6 onwards. In the final two years trainees should spend more time in their area of special interest including advanced fellowship training. The SAC must prospectively approve these posts for training.

## Career structure in oral and maxillofacial surgery

Oral and maxillofacial surgery (OMFS) is based on the practitioner having both medical and dental degrees. They must be on the specialist list in OMFS and be on the General Medical Council (GMC) register. Registration with the General Dental Council (GDC) is optional, but in order to train the dental graduates one must also be fully registered with the GDC.

Trainees traditionally have mostly followed the route of dentistry first then medicine though, increasingly medical graduates are following a path through a second degree in dentistry to train in OMFS.

Once the dual degree is obtained, those who studied medicine second, proceed through foundation training (often only one year) into CST and Member of the Royal College of Surgeons (MRCS). Once the MRCS is obtained they are eligible to apply for a post in specialty training in OMFS. Trainees with dentistry as a second degree need to decide if they are likely to practise dentistry outside of OMFS, in which case they will do dental foundation training for one to two years; once the MRCS is acquired, they can apply to a specialty training post in OMFS.

Specialty training in OMFS lasts five years. Trainees may opt to take additional training in one of the Interface Specialty Fellowships including HNSO, cleft lip and palate, and cosmetic and reconstructive surgery.

## Career structure in plastic surgery

The training programme, designed to last an indicative eight years, includes training in areas of special interest. It comprises three stages: initial (CT1 and 2), intermediate (ST3–6) and final (ST7 and 8). Entry to ST3 is through a national recruitment process including a competitive interview against personal specification including successful acquisition of the intercollegiate MRCS (there is no specific plastic surgical Member of the Royal Colleges of Surgeons (MRCS) as there is for ENT). The training is six years and during this time all trainees will be expected to develop competence in all aspects of the specialty. Having completed the syllabus, attained the required levels of competence and passed the Intercollegiate Examination (usually taken from ST6 onwards), the candidate will be eligible to be awarded a Certificate of Completion of Training (CCT).

Increasingly, at the end of training (ST7 and 8) senior trainees are undertaking special interest fellowships (see below), which take between 12 and 24 months to complete and are appointed through advertisement and selection. These will usually result in the deferment of the CCT until this is completed.

Subspecialty fellowship options include: head and neck surgery, aesthetic surgery, burns, ear reconstruction, genitourinary reconstruction, hand surgery, cleft lip and palate, craniofacial, lower limb, oncoplastic breast surgery (in combination with breast surgeons) and skin oncology.

## Career structure in oncology

Currently, in the UK, there are two main types of oncologists concerned with the management of patients with cancer: medical oncologists (MOs) and clinical oncologists (COs). Both see and assess patients with cancer and both specialities are part of the core membership of cancer multidisciplinary teams (MDTs).

Medical oncologists are physicians trained in the use of systemic drug therapies for cancer, either alone or in combination with other treatments. The RCP supervises training for MOs. Completion of both foundation and core training programmes, culminating in full MRCP, is required prior to entry into MO training. Entry is at the ST3 level with a four-year specialist training programme leading, after passing the Specialty Certificate Examination (SCE), to a Certificate of Completion of Training in Medical Oncology.

Clinical Oncologists are trained in both systemic drug therapy and in the use of radiotherapy. Specialist training in CO also demands full MRCP for entry and begins at the ST3 level. Training takes five years and is supervised by the Royal College of Radiologists (RCR). There is a two-part examination leading to Fellowship of the RCR (FRCR): Part 1, usually passed by the end of ST4, covers the basic sciences of oncology and radiotherapy, whereas Part 2, usually passed during the fourth year of specialist training (ST6), is a clinically based exam and covers the practical aspects of assessing patients and delivering radiotherapy and systemic drug therapy. The award of CCT is, for UK trainees, dependent upon passing both parts of the FRCR examination and completing a further minimum period of one year to achieve advanced oncology training and competencies.

## Integrated and advanced fellowships

The Joint Committee on Surgical Training is an advisory body to the four surgical Royal Colleges of the UK and Ireland for all matters related to surgical training and works closely with the surgical specialty associations in Great Britain and Ireland.

Although HNSO is yet to become a recognised specialty, the Joint Committee on Surgical Training and the Specialty Advisory Committees in OMFS, ORL-HNS and PS, through the Training Interface Group (TIG),^4^ jointly have accredited and recognise several national advanced head and neck surgery posts for training. These fellowships are open to trainees in the three specialties who are in a recognised training post and have completed successfully their Intercollegiate Examination. The recognised fellowships are shown in BOX I.

BOX I **TRAINING INTERFACE GROUP ACCREDITED HEAD AND NECK SURGICAL ONCOLOGY FELLOWSHIP PLACEMENTS**

• Northern – Newcastle Hospitals NHS Foundation Trust
• Northwest 1 – Central Manchester University Hospitals NHS Foundation Trust
• Northwest 2 – Pennine Acute Hospitals NHS Trust
• Oxford – Oxford University Hospitals NHS Trust
• West Midlands – University Hospital Birmingham NHS Foundation Trust
• West of Scotland – Glasgow Royal Infirmary and Southern General Hospital
• Yorkshire – Hull and East Yorkshire Hospitals NHS Trust
• South East Thames – Guy's and St Thomas' Hospital NHS Foundation Trust
• Kent-Sussex and Surrey – Queen Victoria Hospital NHS Foundation Trust
• North Trent – Sheffield Teaching Hospitals NHS Foundation Trust

Recently, the Royal College of Surgeons of England (RCSE) has accredited a number of advanced post-CCT head and neck fellowships. These posts are funded by the individual hospitals but are accredited by the RCSE. These include the advanced head and neck surgery fellowship at Guy's and St Thomas’ Hospital and another one at Charing Cross Hospital.^5^

In addition, there are several hospitals that offer further advanced independent post-CCT HNSO fellowships although these are yet to be recognised by accredited bodies. These programmes are currently available in University Hospital Birmingham, Addenbrooke's Hospital Cambridge, Aintree University Hospitals, Liverpool, Nottingham University Hospital, and St George's Hospital in London.

In the European Union, subspecialty training in HNSO remains diverse.^6^ However, the Union of European Medical Specialists has initiated steps to standardise subspecialty training in the EU and this is likely to have an impact on the current training structure in the near future. Currently, it is recommended that trainees applying for head and neck surgical oncology posts have the required additional and adequate training in this subspecialty. This is often an essential or a desired requirement in the job descriptions.

**Recommendation**

• Trainees applying for Head and Neck Surgical Oncology consultant posts should have completed additional training in the subspecialty

### Key points

• Current Educational Programmes consist of an aim, a syllabus, an assessment matrix and a process for evaluation
• Trainees applying for Head and Neck Surgical Oncology posts should have completed additional training in the subspecialty
• Interface Surgical Fellowships and advanced Post CCT Fellowships are currently available in the UK as part of additional training in Head and Neck Surgical Oncology
• In Clinical and Medical Oncology there are no dedicated Fellowships for additional training.
